# Examining a staging model for anorexia nervosa: empirical exploration of a four stage model of severity

**DOI:** 10.1186/s40337-017-0155-1

**Published:** 2017-11-27

**Authors:** Sarah Maguire, Lois J. Surgenor, Daniel Le Grange, Hubert Lacey, Ross D. Crosby, Scott G. Engel, Kirsty M. Fromholtz, Bryony Bamford, Stephen Touyz

**Affiliations:** 1 Centre for Eating and Dieting Disorders, University of Sydney, Sydney, Australia; 2 Department of Psychological Medicine, University of Otago at Christchurch, Christchurch, New Zealand; 3 Department of Psychiatry, University of California, Christchurch, USA; 4 St Georges University of London, Eating Disorders Research Group, London, UK; 5 Neuropsychiatric Research Institute, School of Medicine and Health Sciences, University of North Dakota, Fargo, North Dakota USA; 6 The London Centre for Eating Disorders and Body Image, London, UK; 7 School of Psychology, University of Sydney, Sydney, Australia

**Keywords:** Anorexia nervosa, Staging, CASIAN, Severity, Stages of illness, Prognosis

## Abstract

**Background:**

An illness staging model for anorexia nervosa (AN) has received increasing attention, but assessing the merits of this concept is dependent on empirically examining a model in clinical samples. Building on preliminary findings regarding the reliability and validity of the Clinician Administered Staging Instrument for Anorexia Nervosa (CASIAN), the current study explores operationalising CASIAN severity scores into stages and assesses their relationship with other clinical features.

**Method:**

In women with DSM-IV-R AN and sub-threshold AN (all met AN criteria using DSM 5), receiver operating curve (ROC) analysis (*n* = 67) assessed the relationship between the sensitivity and specificity of each stage of the CASIAN. Thereafter chi-square and post-hoc adjusted residual analysis provided a preliminary assessment of the validity of the stages comparing the relationship between stage and treatment intensity and AN sub-types, and explored movement between stages after six months (Time 3) in a larger cohort (*n* = 171).

**Results:**

The CASIAN significantly distinguished between milder stages of illness (Stage 1 and 2) versus more severe stages of illness (Stages 3 and 4), and approached statistical significance in distinguishing each of the four stages from one other. CASIAN Stages were significantly associated with treatment modality and primary diagnosis, and CASIAN Stage at Time 1 was significantly associated with Stage at 6 month follow-up.

**Conclusions:**

Provisional support is provided for a staging model in AN. Larger studies with longer follow-up of cases are now needed to replicate and extend these findings and evaluate the overall utility of staging as well as optimal staging models.

**Electronic supplementary material:**

The online version of this article (doi:10.1186/s40337-017-0155-1) contains supplementary material, which is available to authorized users.

## Plain English summary

Anorexia nervosa (AN) is an illness with a broad spectrum of severity. Accessing and triaging treatment matched to stage of illness remains a problem for people with the illness, hence much attention has been paid to the development of a model to stage the illness based on the severity of symptoms. In this study, the severity score provided by a standardised clinical interview assessing the key symptoms of AN, was used to determine stage of illness (stage 1 through to 4) across the AN spectrum, and then examined for the validity of the stages and their ability to predict outcome in 171 people. The stage scores derived from the instrument could differentiate between mild and more severe forms of illness and did predict short-term outcome. Further research in larger samples is required.

## Background

The use of clinical staging as a method for operationalising severity is widespread in the medical disorders including malignancies, cardiac failure, autoimmune diseases and burns to name a few [1] (See Maguire et al., in Latzer, 2010 for review). It is a proven strategy in these disorders where both prognosis and treatment are guided by stage [2]. This is because clinical staging has been argued as a more refined form of diagnosis [3, 4] allowing an individual to be placed at any point in time along a continuum of illness and defining the extent of illness at that time point [5]. Staging can also frame an illness in such a way as to naturally highlight opportunities for early intervention to prevent illness progression, and to match treatments to stages of severity.

In recent years a growing group of clinicians and researchers have argued for staging to be adopted as part of the diagnostic system for mental illnesses [3, 4, 6]. As a result, a number of models of staging of mental disorders have been tentatively proposed, including those for mood disorders [7, 8], panic disorder [9] alcohol use disorders [10], and schizophrenia [11]. Four stage models of illness severity in mental illness are the norm (for a recent systematic review see Cosci and Fava [12], where four stage models for schizophrenia, uni and bipolar depression, alcohol use and panic attacks are summarised). Arguably the field of staging has been influenced by the seminal and most successful disease staging model – the Tumour, Node, Metastatsis (TNM) for cancer, utilising a four stage model roughly equating to mild, moderate, severe and extremely severe stages.

Anorexia nervosa (AN) is often referred to as an illness of varying levels of severity expressed on a continuum [13–19]. Indeed, a growing body of research suggests that the current categorical diagnostic system for eating disorders reflects neither the clinical reality [20–23] nor does it provide meaningful information about severity of illness or prognosis [24, 25]. Thus, clinical staging can be viewed as a middle ground solution between a categorical and dimensional view of illness.

However there have been limited attempts to develop empirically supported measures and models of severity and how, or if, this may be operationalised within a staging model. The empirical literature on AN generally provides insight into which symptoms may be candidate variables contributing to severity. For example, key to the notion of severity is the conferring of a poorer outcome or prognosis for an individual as the illness increases in its ‘severity’. Not all features of AN have been appropriately examined for their prognostic value, but there are a number of key symptoms in AN that have been found to be associated with poorer outcome or prognosis, including body weight and weight history [26–28], duration of illness [27, 29], age at onset [27], drive for thinness [30, 31], restrictive eating [32], body image disturbance [33, 34], motivation for change [35], depression [36, 37], obsessionality [27, 33], purgative behaviours [27], menstrual function [38, 39] & medical complications [40, 41].

To our knowledge, only one other partly validated instrument exists that explicitly attempts to assess AN illness severity. The Short Evaluation of Eating Disorders (SEED) by Bauer et al. [42] assesses severity, rating AN on a 4 stage model from ‘not present’ (0) to ‘extreme’ (3), based on the three DSM-IV criteria of AN – weight, fear of weight gain and distortion of body image, but does not allow for a comprehensive evaluation of all of the clinical features purported to contribute to severity as described above. While these three (one physical, and two psychological) symptoms are undeniably central to the illness, data which differentiates these three symptoms from all others as the strongest indicators of severity is lacking.

To address this need for a method to assess the fuller range of AN symptomatology along the continuum of severity, our group developed an instrument to assess symptoms of AN commonly associated with prognosis and outcome [43]. The full development of the instrument has been published elsewhere [43] and is described in Additional file 1: including the seven illness domains it assesses. Using this empirically developed measure of severity (Clinician Administered Staging Instrument for Anorexia Nervosa (CASIAN) [43]), in this paper we undertake the first exploratory analysis of a staging model for AN, to determine if there is evidence to support further investigation of this conceptualisation in AN. As a conservative approach in applying staging to a novel area, our group have proposed a four stage model, in-line with all prior staging models in mental health and previous attempts in this illness group (described above). As a result we conceptualised the spectrum of AN-like disorders along a continuum of severity with Stage 1 signifying mild or incipient AN, Stage 2 moderate AN, Stage 3 severe AN and Stage 4 extremely severe AN [43, 44]. Utilising CASIAN severity scores we use receiver operating curve (ROC) analysis to empirically derive cut-off scores for a four-stage model of illness. Further, we undertake preliminary exploration of the concurrent validity of the ‘stages’ and existing markers of illness severity such as treatment intensity and diagnosis, as well as explore movement between stages over a six month period in a clinical population.

Specifically, we hypothesise that more severe stages of illness will be associated with more intensive treatment modalities and less severe stages of illness with less intensive treatment. Further, given the more stringent criteria around extent of weight loss, and menses loss in DSM-IV-R full-criteria AN, along with existing data to suggesting AN and DSM-IV-R EDNOS are an a continuum with AN representing the severe end [24, 45], we hypothesise that participants with more severe stages of illness are more likely to meet full-criteria, than those with milder stage illness.

## Method

### Procedure

Participants (*n* = 171) were from the original cohort extensively described elsewhere in the development of the CASIAN [44] including the description of recruitment sites and recruitment method. To recruit a sufficient sample with 6-month follow-up (Time 3) data and reduce participant burden, the original data collection for the CASIAN involved a cross sectional (*n* = 68) and a longitudinal (*n* = 103) condition. Information was not collected on the number or nature of persons who refused to participate in the study.

DSM-IV-R diagnostic criteria were used to identify individuals with AN eligible for participation in the study as this was the diagnostic system version in common use in recruitment sites at that time. To capture the full spectrum of illness severity including persons in partial recovery or in the early stages of illness not yet meeting full criteria, individuals with EDNOS were included in the study. Ricca et al. [21] adjusted DSM-IV criteria for EDNOS -Anorexia Nervosa subtype (EDNOS-AN) were used to determine eligible for the study. That is, these particular participants met all criteria for AN except criterion D (EDNOS-AN(m)), and/or all criteria for AN except criterion A (EDNOS-AN(w)). All subjects were diagnosed by the Primary Clinicians at each site following routine interview and assessment. For reference purposes, and interpretability of data in the light of changes to the diagnostic system, all participants in the study were reclassified according to DSM 5 diagnostic criteria. As both the percentage weight criteria and the menstrual criteria for AN were removed in DSM 5, it should be noted that all 171 participants in this study meet full criteria for AN under the DSM 5 system. In demographic tables both classification systems are reported for the reference of the reader. All analysis of the data retains use of the diagnostic system in use at the time of data collection, DSM-IV-R.

In the cross sectional condition, the participant’s Primary Clinician completed a Clinician Rating of Illness Severity on a 5 point likert scale from 0 to 4, where zero indicated no illness and 4 indicated extremely severe illness. Those in the longitudinal condition were readministered the CASIAN a further 2 times at 3 month follow-up (Time 2) and 6 month follow-up (Time 3). No data from the Time 2 collection is used here as 3 months was deemed too short a time frame for this analysis. We report only on data collected at Baseline (Time 1) and 6 month follow-up (Time 3). The research was reviewed and approved by the relevant Ethics Committees at each recruitment site, and all participants gave informed consent.

### Participant characteristics

The mean age of the total sample was 24.39 years (*SD* = 8.05; range = 16–58), with a mean Body Mass Index (BMI = kg/m^2^) at admission into the study of 16.46 (*SD* = 2.32; range = 9.47–23.63). The mean duration of illness for the total sample was 7.97 years (*SD* = 7.55; range = 0–38). 43.3% of participants met DSM-IV-R full-criteria for AN, 56.7% met Ricca et al., [25] adjusted criteria for EDNOS-AN. Of these 23.4% failed to meet the menstrual criteria and the remaining 33.3% failed to meet the weight criteria. Approximately half (50.9%) of the sample were characterised as having an anorexic illness of the restricting variety, while 49.1% were classified as the binge/purging type [46]. Table 1 shows the characteristics for the study sample as a whole and the samples from the two study arms, inclusive of reference to comparable classification status under DSM 5 diagnostic criteria.

**Table 1:** Characteristics of the study sample

	Total sample	Study sample 1 (Longitudinal)	Study sample 2 (Cross-sectional)
	Mean (SD)	Mean (SD)	Mean (SD)
Age (yrs)	24.39 (8.05)	25.34 (8.63)	22.96 (6.89)
BMI	16.46 (2.32)	16.08 (2.16)	17.03 (2.46)
Duration of Illness (yrs)	7.97 (7.55)	8.69 (7.95)	6.85 (6.78)
DSM 5 AN	100%	100%	100%
DSM-IV-R Full Criteria AN (%)	43.3%	46.6%	38.2%
EDNOS-AN(m) (%)	23.4%	26.2%	19.1%
EDNOS-AN(w) (%)	33.3%	27.2%	42.7%
Restricting subtype (%)	50.9%	51.5%	50%
Binge/Purging subtype(%)	49.1%	48.5%	50%

### Measures

The CASIAN is a 34 item clinician administered interview that assesses seven general domain areas: weight/weight history, onset and duration of illness, dietary control, compensatory behaviours, psychological status (including depression, obsessionality and motivation for change), physical status and egosyntonic features. Twenty-three items compute a validated severity score [43]. (see Additional file 1: for sample items).

Clinician Rating of Severity: In the absence of a validated instrument other than the CASIAN to assess illness severity at the time of data collection, and the availability solely of instruments that were validated either to diagnose only (i.e., categories as ‘ill’ or not ‘ill’) or to assess the extent of a single or several features in AN, an expert clinician rating of whole of illness severity, was chosen as an anchor point for examining stages in the first instance. This type of ‘severity rating’ forms routine practice at specialist centres recruiting to the trial. The Clinician Rating of Severity is a Primary Clinicians assessment of the severity of the person’s AN on a 5 point likert scale from zero (0) through to four (4) where zero represents no illness and four represents extremely severe illness. The extremes and mid-point of the scale have anchor points describing features of an illness at this level of severity, to guide the respondent in their choice. All clinicians participating in the study were highly experienced experts in AN (all >20 years experience). The Clinician Rating of Severity was significantly correlated with scores on the CASIAN at baseline (*r* = .431, *p* < .01) and Patients Rating of their Severity of Illness on the same likert scale (*r* = .417, *p* < .01) (see Additional file 2: for copy of rating scale).

### Statistical analysis

The utility of dividing total scores on the CASIAN into stages of illness according to severity was examined by receiver operating curve (ROC) analysis assessing the relationship between the sensitivity and 1-specificity of each stage of the CASIAN. Chi-square assessed the concurrent validity of each stage against treatment intensity categories and AN sub-types, and explored movement between stages over time. Post-hoc adjusted residual analyses were conducted to explore the relative contribution of cells to the chi square analysis.

All data were analysed using SPSS version 23.

## Results

### Overview

The data analytic plan involved several sequential steps. First, appropriate stage cut-off scores were established, and the total sample divided according to stage. Thereafter we explored the concurrent validity of the staging model by examining the relationships between the proposed stages and existing markers of illness severity – intensity of treatment modality and DSM-IV-R diagnosis. Finally short-term predictive validity of the stages was examined by assessing stage of illness at baseline and 6-month follow-up.

### Sensitivity and specificity analysis

To explore appropriate cut-off scores for each stage of illness, the ratings for all cases in the cross-sectional condition (*n* = 68) that had a Clinician Rating of Illness Severity (*n* = 67), were divided into stages 1 (.5 through 1.49), 2 (1.5 through 2.49), 3 (2.5 through 3.49), and 4 (3.5+). A ROC analysis was then run to determine the best cut-points for total scores in distinguishing stage 1 vs. 2,3, 4 (cut point for stage 2), 1, 2 vs. 3, 4 (cut point for stage 3), and 1, 2, 3 vs. 4 (cut point for stage 4).

**Fig. 1:**
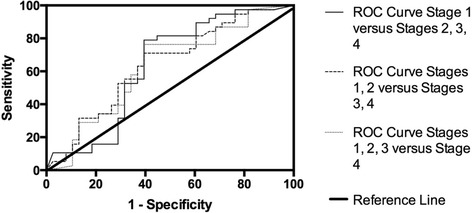
ROC curve for CASIAN score distinguishing between stages

Figure 1 and Table 2 show the ROC curve results for distinguishing the stages. The ROC curve shows the sensitivity (% of those rated as another stage by clinicians at or above the CASIAN Total Score cutoff) and specificity (% of those rated as the desired stage by clinicians below the CASIAN Total Score cutoff) for every possible CASIAN score. The area under the curve (AUC) for the ability of the CASIAN to distinguish Stage 1 from all other stages was 0.678 suggesting that the CASIAN is better than chance at distinguishing individuals at Stage 1 illness and has at least some ability to discriminate between Stage 1 and the other Stages of illness. However this finding did not reach significance (*p* = 0.064).

**Table 2:** ROC curve results

Area under the curve			Coordinates of the curve		
Stage	Area	Sig	Positive if ≥ to	Sensitivity	1-Specificity
1 vs 2,3,4	0.678	0.064	34.50	.929	.455
1,2 vs 3,4	0.706	0.0004	48.50	.694	.290
1,2,3 vs 4	0.674	0.070	52.50	.545	.304

The AUC for ability of the CASIAN to distinguish Stage 1 and 2 from Stages 3 and 4 (i.e. the cut-off point for Stage 2) was 0.706, reaching (*p* = 0.004). This finding would suggest that the CASIAN is better than chance at distinguishing individuals at Stage 2 illness and has the ability to discriminate between Stage 2 and the other Stages of illness.

The AUC for ability of the CASIAN to distinguish Stage 1, 2, 3 from Stage 4 illness (i.e. the cut-off score for stage 3) was 0.674 with *p* = 0.070. This finding would suggest that the CASIAN has at least some ability to discriminate between Stage 4 and the other Stages of illness. Although approaching significance the test did not reach it suggesting the CASIAN is not statistically significantly better than making this decision based on pure chance.

Table 2 shows the curve coordinates (sensitivity and specificity) for each of the three cut-off points for total CASIAN scores selected in the sample of 67 cases with Clinician Ratings of Illness Severity. To maximise sensitivity and specificity of the instrument for identifying each stage a cut-off score on the CASIAN of 34.50 as the lower limit for Stage 2 was selected, 48.50 as the lower limit for Stage 3 and 52.5 as the lower limit for Stage 4.

### Frequency of stages

To explore the potential utility of stages within AN, the above determined cut-off points suggested by ROC were then applied to the entire original cohort (*n* = 171).

### Validity of the proposed stages of illness

**Concurrent Validity:** Stage of illness and treatment modality

**Fig. 2:**
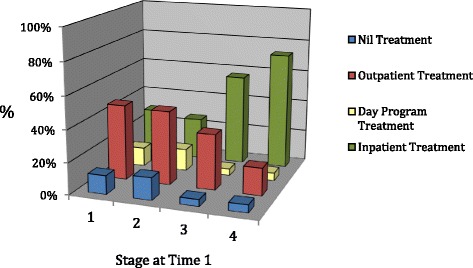
Stages of illness and types of treatment

Figure 2 depicts the relationship between ‘stage’ of illness and intensity of treatment modality. The percentages of participants at different stages of illness according to the treatment type they are engaged in is also outlined in Table 3. Figure 2 shows that for the milder stages of illness (Stage 1 and 2) a greater percentage were engaged in less intensive treatment (outpatient therapy) as depicted by the elevated red bars in these stages. For those in the more severe stages of illness (Stage 3 and 4) a greater percentage were engaged in more intensive treatment (inpatient therapy), as indicated by the elevated green bars in these stages. A chi square analysis of the differences in treatment modality utilised by individuals at different stages of illness was found to be significant*, (χ* ^2^ [df = 9, *n* = 171] =32.47, *p* = .000). A post hoc test using the adjusted residual method [47, 48] was conducted to determine which cells made the largest contribution to the significant chi square, *p* values were calculated for each adjusted residual (z score) and residuals adjusted for the type 1 error rate (∝/16 as 16 cells being tested) producing a significance level of ∝ = .00031.

**Table 3:** Relationship between stage 1 and treatment modality

		Treatment type			
		Nil	Outpatient	Day program	Inpatient
Stage 1	Count	2	8	2	5
	%	11.8	47.1	11.8	29.4
	Adj. Resid.	0.6	1.5	0.6	−2.0
	*P* value	0.55	0.13	0.55	0.05
Stage 2	Count	7	23	7	13
	%	14	46	14	26.0
	Adj. Resid.	1.8	2.7	1.8	−4.5
	*P* value	0.07	0.01	0.07	**0.00**
Stage 3	Count	1	8	1	13
	%	4.3	34.8	4.3	56.5
	Adj. Resid.	−0.7	0.4	−0.7	0.4
	*P* value	0.48	0.69	0.48	0.69
Stage 4	Count	4	14	4	59
	%	4.9	17.3	4.9	72.8
	Adj. Resid.	−1.5	−3.7	−1.5	5.0
	*P* value	0.13	**0.00**	0.13	**0.00**

Using this significance level, in Table 3 the *p* value from three cells (bold, underlined) reach significance. For those individuals in Stage 2 illness there was a significantly smaller number receiving inpatient treatment. For those in Stage 4 illness the pattern was reversed with significantly more in inpatient treatment and less in outpatient.

**Concurrent validity:** DSM-IV-R diagnosis and stage

**Fig. 3:**
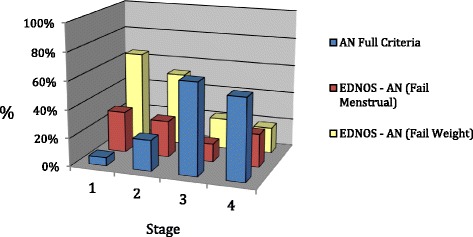
Stage of illness and diagnosis

The relationship between stage of illness and DSM-IV-R diagnosis was also examined. Figure 3 depicts the overall pattern of results when the sample was divided according to the stage of illness and primary eating disorder diagnosis at the time of first assessment.

As the figure and percentages in Table 4 suggest, those individuals in the milder stages of illness (Stage 1 and 2) tended to be diagnosed as DSM-IV-R EDNOS cases, and the majority failed to meet the weight criteria, as indicated by the elevated yellow bars in these stages. Whereas, participants in the more severe stages of illness (Stage 3 and 4) tended to be diagnosed with full syndrome AN, as indicated by the elevated blue bars in these stages. A chi square analysis of the differences in diagnoses across the Stages of Illness at first assessment was significant*, (χ* ^2^ [df = 6, *n* = 171] =35.322, *p* = .000). A Post hoc test of the adjusted residuals was conducted, *p* values were calculated for each adjusted residual (z score) and residuals adjusted for the type 1 error rate (∝/12 as 12 cells being tested) producing a significance level of ∝ = .00042.

**Table 4:** Relationship between stage and diagnosis

		Diagnosis		
		AN	EDNOS - AN (Fail Menstrual)	EDNOS - AN (Fail Weight)
Stage 1	Count	1	5	11
	%	5.9	29.4	64.7
	Adj. Resid.	−3.3	0.6	2.9
	*P* value	**0.00**	0.55	**0.00**
Stage 2	Count	11	13	26
	%	22	26	52
	Adj. Resid.	−3.6	0.5	3.3
	*P* value	**0.00**	0.62	**0.00**
Stage 3	Count	15	3	5
	%	65.3	13.0	21.7
	Adj. Resid.	2.3	−1.3	−1.3
	*P* value	0.02	0.19	0.19
Stage 4	Count	47	19	15
	%	58	23.5	18.5
	Adj. Resid.	3.7	.0	−3.9
	*P* value	**0.00**	1.00	**0.00**

Utilising this significance level, in Table 4, we can see that the *p* value from six cells reached significance (bold, underlined). For those individuals in the milder stages (Stage 1 and 2), there was a significantly smaller number who met full-criteria DSM-IV-R AN and significantly larger proportions diagnosed with EDNOS-AN (fail weight criteria). In the most severe stages of illness (Stage 4) significantly more were diagnosed with DSM-IV-R full-criteria AN and less with EDNOS-AN (fail weight criteria).

**Predictive Validity:** Baseline Stage of Illness compared to 6-month follow-up

Finally movement between stages of illness from baseline to 6 month follow-up was explored for those participants in the longitudinal condition (*n* = 103) who completed a staging assessment at 6 month follow-up (*n* = 74).

**Fig. 4:**
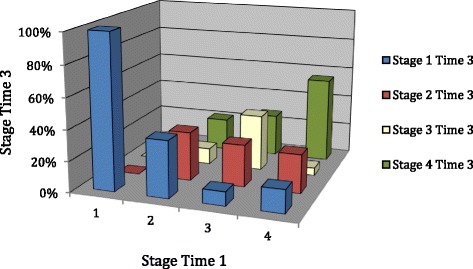
Stage of illness at baseline (Time 1) and 6 month follow-up (time 3)

Figure 4 depicts these results, with the percentages also outlined in Table 5. Individuals in Stage 1, 3 and 4 were more likely to still be classified within the same stage at 6 month follow-up. Stage of Illness at Time 3 was significantly predicted by Stage of Illness at Time 1 *(χ* ^2^ [df = 9, *n* = 74] =28.86, *p* = .001). A post hoc test using the adjusted residual method was conducted, *p* values were calculated for each adjusted residual (z score) and residuals adjusted for the type 1 error rate (∝/16 as 16 cells being tested) producing a significance level of ∝ = .00031.

**Table 5:** Movement across stages from time 1 to time 3

		Stage at Time 3 (6 months FU)			
		Stage 1	Stage 2	Stage 3	Stage 4
Stage 1	Count	4	0	0	0
	%	100	0	0	0
	Adj. Resid.	3.6	−1.2	−0.7	−1.7
	*P* value	**0.00**	0.23	0.48	0.09
Stage 2	Count	7	6	2	4
	%	36.8	31.6	10.5	21.1
	Adj. Resid.	1.5	0.7	0.0	−1.9
	*P* value	0.13	0.48	1.00	0.06
Stage 3	Count	1	3	4	3
	%	9.1	27.3	36.4	27.3
	Adj. Resid.	−1.3	0.1	3.0	−0.9
	*P* value	0.19	0.92	**0.00**	0.37
Stage 4	Count	6	10	2	22
	%	15	25	5	55
	Adj. Resid.	−2.0	−0.1	−1.7	3.0
	*P* value	0.05	0.92	0.09	**0.00**

Utilising this significance level, from Table 5, three cells reached significance (bold, underlined). For those individuals in Stage 1, 3 and 4 there was a significantly larger proportion in the same Stage of Illness at 6 month follow-up.

## Discussion

While several AN staging models have been proposed, as far as we are aware our work goes one step further to empirically examine staging in a dataset of people with AN and explore if this conceptualisation of illness has potential. Despite the relatively large sample size for a rare illness, the present examination needs be regarded as exploratory in nature, examining the potential utility of a provisional model. As is the case with all innovations, this study is intended to stimulate further testing of the model through both replication and extension within further large samples. We especially encourage other independent research groups to empirically scrutinise the model.

While a four-stage model (derived from CASIAN scores) may possess some ability to detect cases at different stages of illness, only one of the three stage cut-off investigations reached significance. That is, between milder stages of illness 1–2 and more severe stages 3–4.The ability to distinguish Stage 1 from all others and Stage 4 from all others still warrants further investigation with larger and ideally multi-site studies.

As a way of investigating if the proposed staging model correlated with existing markers of illness severity (like intensity of treatment and full-syndrome versus partial-syndrome diagnosis) it was hypothesised that people with more severe stages of illness would engage with more intensive treatment options and the study found some support for this. The residual analysis suggested the largest contributions to this were in persons with the most severe illness (Stage 4), who were significantly more likely to be engaged in hospital treatment and less likely to be in outpatient care. In addition there was some evidence to suggest that persons at a milder stage of illness, Stage 2, were significantly less likely to be in inpatient care. The failure to find significant contributions from other stages of illness may be a result of sample size limitations, because recruitment was most successful through treatment facilities this sample has a larger number of participants in the more severe stages of illness. Alternatively, it could reflect the lack of treatment options available to many individuals with AN.

As hypothesised, support was found for stages of illness to reflect DSM-IV-R diagnoses in that people in the milder stages of illness, both Stage 1 and 2, were more likely to be diagnosed with DSM-IV-R nosology EDNOS, and those in Stage 3 and 4 illness more likely to be assigned full-criteria diagnosis. Other studies have confirmed such an understanding of DSM-IV-R EDNOS-AN cases. For example, a large meta-analytic review comparing EDNOS and the full syndrome ED cases also concluded that full-syndrome AN represents the severe end of a continuum that is likely to include EDNOS illnesses at the milder end of this [24]. It should be noted that application of DSM-5 diagnostic criteria to participants in this study saw all redefined as full-syndrome AN according to DSM 5.

Successful staging in AN would have a number of clinical and research uses. To be able to assess any presenting individual against a number of empirically derived symptoms known to accurately assess severity and predict prognosis would have uses in treatment design, matching patients to treatment, improving the client and carer experience and understanding of the illness and the likely outcome, as well as enabling streamlined research of participants at varying stages of illness. Ideally with further research, a refined subset of core symptoms could be identified to stage an individual efficiently, match them with suitable treatments and predict with some accuracy their prognosis, therefore the range of variables examined in this study need further examination and refinement to determine which illness factors are the best markers and prognostics for the most efficient staging model.

The ultimate utility of a staging model is arguably to be able to predict outcome. This needs to be done over the longer term ideally with assessment and ‘staging of illness’ at the earliest possible point in presentation and assessment of outcome over the long term. As a preliminary exploration of a staging model and its relationship to outcome, we found that, for those individuals in Stage 1, 3 and 4, there was a significantly larger proportion in the same Stage of Illness at 6 month follow-up. This is a very short follow-up period, and not one with participants first assessed immediately after onset, so this finding may well reflect that the time frame for assessment is too short to assess illness trajectory. Further examination of the prognostic utility of stages is worth further investigation in large samples, over long follow-up periods.

### Limitations

There are a number of limitations to the study, the first of which is the need for a larger concurrently recruited sample with a more even spread of illness severity. Obtaining sufficient sample sizes of rare disorders is always difficult and AN is an especially difficult population to recruit. The recruitment strategy also introduced a sampling bias in that people not receiving treatment were underrepresented and it was not possible to control for treatment and illness duration factors. Importantly therefore, the staging concept should be explored capturing those in the community, in very early in the illness trajectory, and then followed over a much longer term. Inevitably, these may need to be multisite collaborations and these are feasible..Finally in terms of sampling, there may also have been an implicit bias amongst those participants who agreed to participate in the study or agree to long follow-up. For example, hospitalised participants who more likely to agree to 6 months follow-up may have been more welcoming of the diversion (of participation) as opposed to those receiving outpatient treatment who may have wanted less intrusion into their life.

A second major limitation relates to the lack of any ‘gold standard’ severity measures in AN at the time of data collection in which to develop stage cut offs We needed to develop a clinician rating of severity using highly experienced clinicians but this was was deemed superior to any existing ill/not ill diagnostic instrument or existing instruments that measure single dimensions of illness. Cut-off for stages using the CASIAN require examination using other measures of illness severity, and further studies need to expand upon the examination of a staging model using other anchor points, and other statistical methods.

## Conclusion

While the limitations of this study need to be addressed in studies with larger consecutively recruited samples, provisional support for the conceptualisation of illness stages within the AN illness continuum was found here. We were able to demonstrate an instrument to distinguish between milder and more severe stages of illness and then relationships between these stages and relevant illness factors. The current data can only be considered as highly provisional but encouraging of further studies in an area struggling with slow advances in improving treatment outcomes. Capturing key aspects of illness staging is one of the identified key goals in order to help psychiatry potentially benefit from pathway models proven so useful with some medical conditions [49, 50]. Time will tell if psychiatric classifications systems can be enhanced by atriculating stages found to be clincially useful. 

## Additional files

Additional file 1: Sample items from each dimension of the clinician administered staging instrument for anorexia nervosa (CASIAN). (DOC 29 kb)

Additional file 2: Staging anorexia nervosa study. Clinician rating of severity. (DOC 45 kb)

## Abbreviations

AN: Anorexia Nervosa
AUC: Area under the curve
BMI: Body mass index
CASIAN: Clinician Administered Staging Instrument for Anorexia Nervosa
DSM: Diagnostic and statistical manual of mental disorders
EDNOS: Eating disorder not otherwise specified
ROC: Receiver operating curve
