# Supplementary material for: Examining a staging model for anorexia nervosa: empirical exploration of a four stage model of severity
Source: J Eat Disord. 2017 Nov 27;5:41. doi: 10.1186/s40337-017-0155-1 (PMC5702958; doi:10.1186/s40337-017-0155-1)
Supplement: Supplementary file 1 — Sample items from each dimension of the clinician administered staging instrument for anorexia nervosa (CASIAN). (DOC 29 kb) [file 40337_2017_155_MOESM1_ESM.doc]

Appendix A

Development of the CASIAN and sample items

In an earlier paper [43] we outlined the development process of the CASIAN in detail. Briefly, potential items for the CASIAN were developed in three phases; domain generation, content generation, and item generation. Thereafter the psychometric properties of the CASIAN were then evaluated in a sample 171 participants with DSM-IV AN or subthreshold AN classed in the Eating Disorder Not Otherwise Specified (EDNOS) category. Item analysis procedures and factor analytic studies were conducted yielding a 6 factor structure for the instrument, which were labelled ‘Motivation’, ‘Weight’, ‘Illness Duration’, ‘Obsessionality’, ‘Bulimic Behaviours’ and ‘Acute Issues’. A 23 item validated severity score computed from the CASIAN was established and the CASIAN demonstrated high reliability as assessed by internal consistency (α = .811), test-retest reliability (*r* = .957) and inter-rater reliability (*r* = .973). Evidence of its convergent validity with other eating disorder symptom questionnaires, concurrent and discriminant validity as well as short-term predictive validity was also demonstrated.

Table A

Sample items from each Dimension of the Clinician Administered Staging Instrument for Anorexia Nervosa (CASIAN)

**______________________________________________________________**

Stage Dimension

______________________________________________________________

Weight/Weight History

*Item: Where does the persons current BMI fall?*

0 No Illness At or above 20

1 Mild AN At or above 17.5 but less than 20

2 Moderate AN At or above 15 but less than 17.5

3 Severe AN At or above 13 but less than 15

4 Extremely Severe AN Less than 13

- - - - - - - - - - -- - - - - - - - - - - - - - - - - - - - - - - - - - - - - - - - - - - - - - - - - - - - - - - - -

Restriction

*Item: Average Daily Caloric Intake*

0 No Illness All food groups represented in adequate quantities (3 meals & snacks). Average daily caloric intake of about 2100

1 Mild AN Reduced quantities/one food group restricted (e.g. high fats), snacks missing, 3 main meals present. Average daily caloric between 1700 and 2100

2 Moderate AN Significantly reduced quantities or one food group excluded from diet. Average daily caloric intake of between 1000 and 1700

3 Severe AN Very limited quantities or a no. of food groups eliminated, restricted variety of foods. Average daily caloric intake of between 500 and 1000

4 Extremely Severe AN Severely limited quantities and variety small quantity of liquid. Average daily caloric intake of less than 500

- - - - - - - - - - -- - - - - - - - - - - - - - - - - - - - - - - - - - - - - - - - - - - - - - - - - - - - - - - - -

Binge/Purge Behaviours

*Item: Self-Induced Vomiting*

0 No Illness The person does not induce vomiting

1 Mild AN The person induces vomiting rarely

(once a month or less).

2 Moderate AN The person induces vomiting about once a week

3 Severe AN The person induces vomiting on a daily basis

4 Extremely Severe AN The person induces vomiting after every meal

- - - - - - - - - - -- - - - - - - - - - - - - - - - - - - - - - - - - - - - - - - - - - - - - - - - - - - - - - - - -

Motivation

*Item: Acceptance of Normal Weight*

0 No Illness The person is working hard to maintain at their minimum healthy weight

1 Mild AN The person is putting in a lot of effort to reach their minimum healthy weight

2 Moderate AN The person has decided that they will attempt to reach their minimum healthy weight

3 Severe AN In some ways they can see that they may be better off if they were at there minimum healthy weight

4 Extremely Severe AN As far as they are concerned they do no need to be at their minimum healthy weight

- - - - - - - - - - -- - - - - - - - - - - - - - - - - - - - - - - - - - - - - - - - - - - - - - - - - - - - - - - - -

Chronicity

*Item: Duration of Pathological Weight Losing Behaviours*

0 No Illness Less than 1 month

1 Mild AN 1 month or more but less than 6 months

2 Moderate AN 6 months or more but less than 2 years

3 Severe AN 2 years or more up to 5 years

4 Extremely Severe AN More than 5 years

- - - - - - - - - - -- - - - - - - - - - - - - - - - - - - - - - - - - - - - - - - - - - - - - - - - - - - - - - - - -

Obsessionality

*Item: Preoccupation with obsessions around food, weight, shape, fat and body.*

0 No Illness None of the time

1 Mild AN Mild, less than 1 hour per day or occasional intrusion

2 Moderate AN Moderate, 1-3 hours per day or frequent intrusion

3 Severe AN Severe, greater than 3 and up to 8 hours per day or very frequent intrusion

4 Extremely Severe AN Extreme, greater than 8 hours per day or constant intrusion
