# Supplementary material for: Examining a staging model for anorexia nervosa: empirical exploration of a four stage model of severity
Source: J Eat Disord. 2017 Nov 27;5:41. doi: 10.1186/s40337-017-0155-1 (PMC5702958; doi:10.1186/s40337-017-0155-1)
Supplement: Supplementary file 2 — Staging anorexia nervosa study. Clinician rating of severity. (DOC 45 kb) [file 40337_2017_155_MOESM2_ESM.doc]

Appendix 2.

**Staging Anorexia Nervosa Study**

**Clinician Rating of Severity**

Client’s name: ____________________________________________________

Date this form completed: ___________________________________________

Name of Clinician completing form:______________________________________

Please consider your client’s eating disorder as being part of a spectrum of illness severity. Using the anchor descriptions as guides and your clinical judgement, could you please mark where on the continuum below you would consider your client to be at the present time. Please rate current severity only, do not make reference to past symptomatology.

0 1 2 3 4

No illness Significant Illness Extremely Severe Illness

- weight at healthy/resting level - significant weight loss - extreme weight loss

- nil pathological thinking - pathological thinking - extreme/continuos pathological thinking

- nil body image disturbance - body image disturbance - extreme body image disturbance

- menstruating where appropriate - significant medical complications
